# Restrictive Allograft Syndrome After COVID-19 Pneumonia: A Case Report

**DOI:** 10.7759/cureus.54583

**Published:** 2024-02-20

**Authors:** Yuji Ohizumi, Ryo Kurokawa, Shiori Amemiya, Tatsuya Ito, Masaaki Sato, Osamu Abe

**Affiliations:** 1 Radiology, Graduate School of Medicine, The University of Tokyo, Tokyo, JPN; 2 Respiratory Medicine, Ome Municipal General Hospital, Tokyo, JPN; 3 Thoracic Surgery, Graduate School of Medicine, The University of Tokyo, Tokyo, JPN

**Keywords:** steroids, dyspnea, covid-19, bronchiolitis obliterans syndrome, allografts

## Abstract

Chronic lung allograft dysfunction (CLAD) continues to be the leading cause of death in the long term after lung transplantation (LTx). CLAD has the following two main subtypes: bronchiolitis obliterans syndrome (BOS) and restrictive allograft syndrome (RAS). BOS features obstructive lung dysfunction, while RAS features restrictive lung dysfunction. Overall, RAS has a worse prognosis. The pathophysiology of CLAD is not fully understood; however, pulmonary infections can trigger CLAD, including coronavirus disease 2019 (COVID-19) pneumonia. Here, we describe a case of a 55-year-old female who received LTx about seven years ago and developed RAS after COVID-19 pneumonia. RAS was ultimately diagnosed based on the clinical course and imaging findings. Steroid pulse therapy and empirical antimicrobial therapy were initiated, but respiratory failure progressed, and the patient died 139 days after COVID-19 diagnosis, and 83 days after dyspnea progression. Clinicians should be aware of unusual stair-step clinical courses and imaging features in a given setting of pulmonary infection including COVID-19 to suspect CLAD in lung transplant patients.

## Introduction

Chronic lung allograft dysfunction (CLAD) is a complication of lung transplantation (LTx) that is characterized by a progressive decline in lung function [1]. CLAD remains the major cause of death after the first year post-LTx [2]. CLAD has the following two main phenotypes: bronchiolitis obliterans syndrome (BOS) and restrictive allograft syndrome (RAS). BOS, which is associated with obstructive airflow limitation, was described by Cooper et al. in 1993 [3]. RAS was described by Sato et al. in 2011 and is associated with restrictive lung dysfunction [4]. Although the pathogenesis of CLAD is not fully understood, multiple immunological factors are thought to be involved [5]. Pulmonary infection is known to trigger CLAD, and cases of CLAD following severe acute respiratory syndrome coronavirus 2 (SARS-CoV-2) infection have been reported [6-8]. Here, we described a case of RAS following coronavirus disease 2019 (COVID-19) pneumonia.

## Case presentation

**Figure 1:**
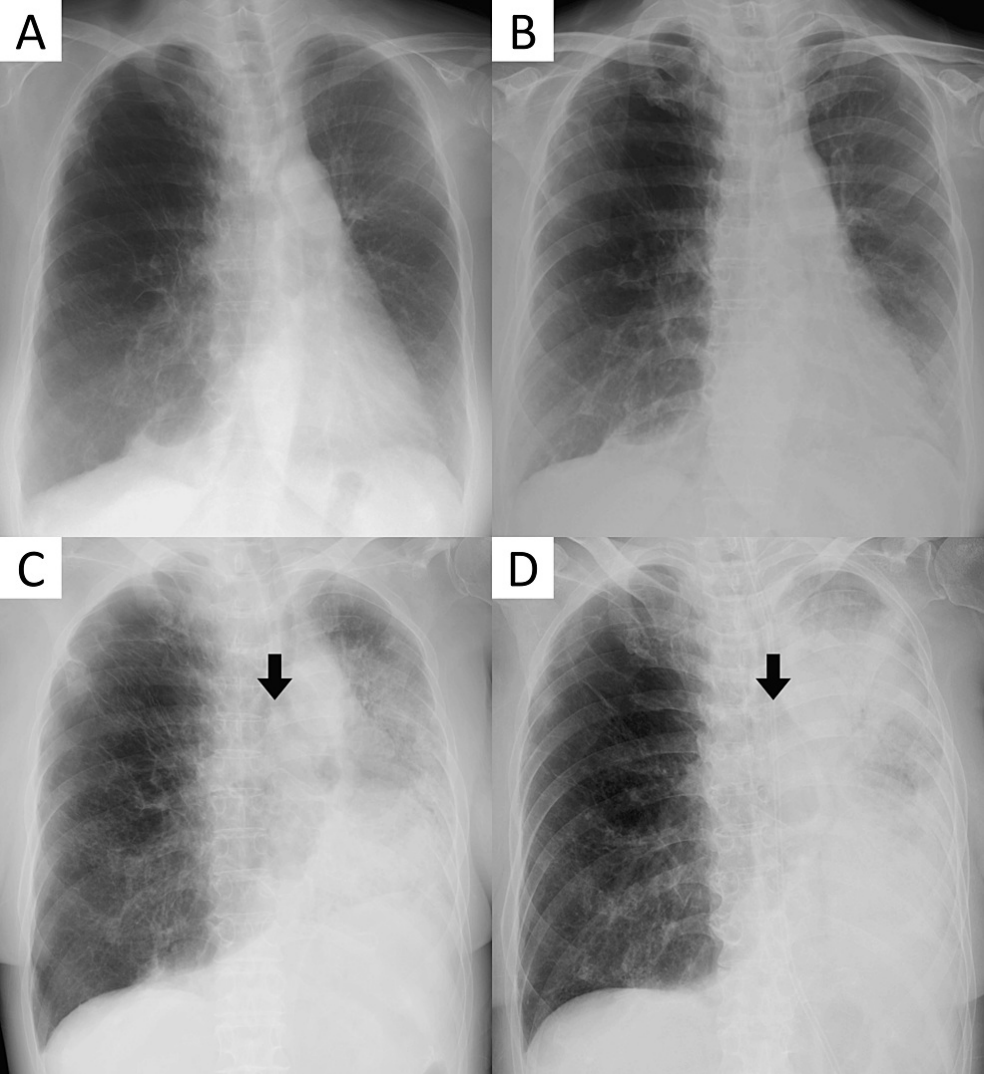
Chest x-ray findings of the patient. (A) Baseline chest x-ray after LTx. The right lung has a large volume and decreased density due to lymphangioleiomyomatosis, and there is no pulmonary infiltrate in the transplanted left lung, (B) diagnosis of COVID-19 pneumonia with increased density in the left middle and lower lung, (C) on the second admission to our hospital - 18 weeks after her COVID-19 diagnosis and 10 weeks after her dyspnea worsened - the pulmonary infiltrates in the left lung progress and mediastinal deviation appears (arrow), and (D) despite the steroid pulse, the left transplant lung opacification, volume loss, and mediastinal deviation (arrow) get worse. LTx: lung transplantation; COVID-19: coronavirus disease 2019

**Figure 2:**
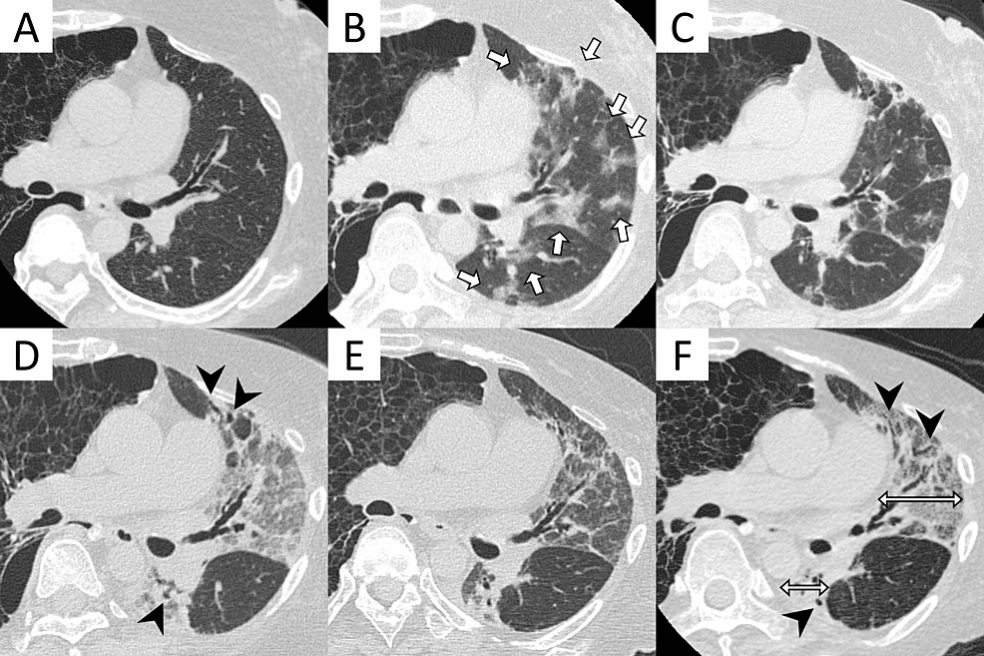
Chest computed tomographies. (A) Baseline CT scan after LTx. The right lung is replaced by cysts due to lymphangioleiomyomatosis, and the left transplanted lung has no radiological abnormality, (B) diagnosis of COVID-19 pneumonia with multiple ground-glass opacities and consolidations (arrows) in the left lung, (C) after treatment for COVID-19, ground-glass opacities and consolidations shrink, (D) with dyspnea progression, broad ground-glass opacities and consolidations appear with traction bronchiectasis (arrowheads) in the left lung, (E) after treatment with a steroid pulse and increased dose of immunosuppressive medication, abnormalities are improved, (F) on the second admission to our hospital - 18 weeks after her COVID-19 diagnosis and 10 weeks after her dyspnea worsened - ground-glass opacities and consolidations with traction bronchiectasis (arrowheads) get worse and the left lung volume decreases (double arrow). This “stair-step” course of lung dysfunction and these typical abnormalities suggest RAS. LTx: lung transplantation; COVID-19: coronavirus disease 2019; RAS: restrictive allograft syndrome

A 55-year-old female who had received left LTx about seven years ago for lymphangioleiomyomatosis presented with fever, diarrhea, upper respiratory symptoms, and mild dyspnea. At 37 years of age, she was diagnosed with pulmonary lymphangioleiomyomatosis and underwent a left LTx at 48 years of age from a brain-dead donor. She underwent pleurodesis for a right pneumothorax after LTx. Since the age of 37, she had not smoked, had consumed alcohol only occasionally, and had been vaccinated for COVID-19 four times. She had no history of tuberous sclerosis complex. She routinely attended our hospital for follow-up visits, and had no major problems after LTx, clinically and radiologically (Figure 1, panel A and Figure 2, panel A).

However, 12 days before admission to our hospital, she presented with fever, diarrhea, upper respiratory symptoms, and mild dyspnea. She was diagnosed with COVID-19 with a positive SARS-CoV-2 polymerase chain reaction (PCR) test at another hospital. Although her symptoms abated within the next three days with symptomatic therapy, the symptoms recurred, and she presented to our hospital. The vital signs were as follows: body temperature 37.9°C, blood pressure 116/66 mmHg, pulse rate 116 beats/min, respiratory rate 16 breaths/min, and oxygen saturation 94% on 0.5 L/min of oxygen via nasal cannula. The result of the SARS-CoV-2 PCR test continued to be positive. Chest x-ray indicated the increased density in the left middle and lower lung, and computed tomography (CT) showed ground-glass opacities and consolidations in the left lung, which were compatible with COVID-19 pneumonia (Figure 1, panel B and Figure 2, panel B). The patient was treated with a combination of remdesivir (200 mg on the first day, followed by 100 mg from the second to the fifth day) and dexamethasone (6.6 mg/day for five days). Fever and respiratory distress improved, leading to her discharge on the 17th day. At the time of discharge, the absolute lymphocyte count was 1.36 × 10^3^/dL.

A week after discharge, mild respiratory distress persisted, although CT indicated improvement in ground-glass opacities (Figure 2, panel C). Four weeks after discharge, her respiratory distress worsened, and she was urgently transported to another hospital. CT revealed new ground-glass opacities and consolidations with traction bronchiectasis in the left lung (Figure 2, panel D). She underwent treatment with a steroid pulse and an increased dose of immunosuppressive medication for two months, which temporarily alleviated the symptoms. CT also showed improvement in ground-glass opacities and consolidations (Figure 2, panel E). She was transferred to a rehabilitation hospital. However, after approximately a week, her respiratory distress worsened, and she presented to our hospital again.

The patient’s vital signs at the second admission were as follows: body temperature 37.5°C, blood pressure 104/70 mmHg, pulse rate 150 beats/min, respiratory rate 28 breaths/min, and oxygen saturation 91% on 10 L/min of oxygen via reservoir mask. The result of the SARS-CoV-2 PCR test turned negative. Chest radiography revealed increased density of the left transplanted lung and leftward deviation of the mediastinum (Figure 1, panel C). CT showed worsening ground-glass opacities and consolidations with traction bronchiectasis, suggesting strong contractile changes and progressive volume loss only in the transplanted left lung (Figure 2, panel F).

The patient was diagnosed with RAS rather than post-COVID-19 pulmonary fibrosis based on the following reasons: her respiratory failure demonstrated a “stair-step” pattern of progression after an initial improvement of SARS-CoV-2 pneumonia, the abnormalities were localized in the transplanted left lung, while the right lung did not show the evidence of fibrosis, there were no other evident causes for pulmonary fibrosis or graft failure, and the transplanted lung showed notable volume reduction over time which is characteristic for RAS.

Steroid pulse therapy and empirical antimicrobial therapy were initiated for RAS. Despite medication, her respiratory condition did not improve, and radiography showed a further decrease in the permeability of the left lung (Figure 1, panel D). Thereafter, respiratory failure progressed unabated, and the patient died 13 days after admission, it was 2572 days after LTx, 139 days after COVID-19 diagnosis, and 83 days after dyspnea progression.

## Discussion

In this study, we described a case of a patient who developed RAS after SARS-CoV-2 pneumonia. Chest radiography and CT showed a decline in the transplanted lung volume. CLAD is a syndrome of persistent decline in lung function after LTx [1] and is known to be the major cause of death after the first year post-LTx [2]. CLAD has two main subtypes known as BOS and RAS, which sometimes occur simultaneously. BOS features airflow obstruction defined as forced expiratory volume in 1 s (FEV1) per forced vital capacity (FVC) <0.70 and RAS features restrictive lung dysfunction defined as a ≥10% reduction in baseline total lung capacity (TLC) or suspected by FVC ≤80% from baseline. A mixed phenotype was defined as a combination of obstructive and restrictive features [9]. Approximately 70% of CLADs are characterized as BOS and approximately 30% are characterized as RAS [4].

One study showed that most CLAD cases were initially diagnosed as either BOS or RAS, and 9.3% developed a mixed phenotype [10]. RAS has a worse prognosis than BOS, with a median survival of 309 days following onset, as opposed to 1070 days for BOS [11]. Imaging findings of CLAD differ between BOS and RAS. BOS exhibits bronchial wall thickening and cylindrical bronchiectasis, representing central airway abnormalities, as well as air trapping and mosaicism of lung attenuation, reflecting peripheral obstructive changes. In contrast, RAS initially shows non-specific ground-glass nodules, septal lines, and focal consolidation, followed by pleural thickening, dense peripheral consolidation, traction bronchiectasis, architectural distortion, and loss of lung volume. These characteristics were consistent with the longitudinal CT imaging findings in the present case. Mixed BOS and RAS exhibit a combination of radiological features [12,13]. Although the pathophysiology of CLAD is not fully understood, several mechanisms, including immunological mechanisms (e.g., innate and adaptive immunity, humoral and cellular immunity, and matrix remodeling), are thought to play an important role [5]. Acute rejection and infection are known risk factors for developing CLAD. Respiratory pathogens can cause CLAD by contracting the inflammatory environment within the transplanted lungs [6]. Several therapies have been employed to treat CLAD, including immunomodulatory therapy, immunosuppressive therapy, and antifibrotic medications. However, re-transplantation is the only therapy that can increase survival in patients with CLAD [9].

In recent years, the association between COVID-19 and CLAD has become recognized. Mahan et al. conducted a single-center observational study among LTx patients surviving COVID-19, particularly regarding respiratory function [7]. Forty-four patients were included, with 18 patients displaying persistent reductions in FVC or FEV1, and 15 patients of them experiencing restrictive or mixed respiratory impairments. Furthermore, three patients met the diagnostic criteria for CLAD, all of whom exhibited the RAS subtype. In comparison to other respiratory viral infections, COVID-19 appeared to induce prolonged respiratory function decline, with more likely restrictive impairments than obstructive ones [7]. Multivariate analysis in their study identified low absolute lymphocyte count (ALC) (less than 0.6 × 10^3^/dL) and elevated ferritin (more than 150 ng/mL) at the time of discharge as independent risk factors for significant respiratory function decline. Although the exact mechanism remained uncertain, they speculated that low ALC and elevated ferritin may be linked to a cytokine storm and serve as consistent biomarkers for predicting allograft dysfunction. In the present case, the serum ferritin levels after COVID-19 treatment were not assessed, and ALC did not decrease, hence not meeting the risk factors outlined in the multivariate analysis. Roosma et al. conducted a retrospective multicenter study investigating the impact of COVID-19 on patients after LTx [8]. Seventy-four patients were included, and there was a respiratory function decline after COVID-19. Subsequently, a gradual improvement of lung function was observed, although residual respiratory function impairment persisted when compared to the pre-COVID-19 baseline. The restrictive pattern of respiratory function impairment was dominant in their patients. Of the 53 non-CLAD patients, six patients newly developed CLAD: three with BOS, and three with RAS. Moreover, one out of the 18 patients initially diagnosed with BOS transitioned into an RAS phenotype. The restrictive impairment pattern was attributed to the pulmonary alveolar damage and interstitial fibrosis induced by COVID-19, with the degree of radiological abnormalities on CT and X-ray thought to be correlated with the extent of respiratory function impairment. Hirawat et al. suggested that cytokine storm might be a mechanism behind the development of fibrosis after COVID-19 [14]. Cytokine storm is defined as the uncontrollable overproduction of pro-inflammatory markers and COVID-19 causes a cytokine storm through various mechanisms such as virus proliferation-induced pyroptosis and neutralizing antibody production via adaptive immunity, leading to multi-organ dysfunction including acute respiratory distress syndrome in the lungs [15]. The hypothesis that cytokine storm is involved in post-COVID-19 fibrosis aligns with the findings of Roomsa et al., those who identified risk factors associated with a cytokine storm for respiratory function decline after COVID-19 [8].

The progressive pattern of RAS differs from that of pulmonary fibrosis after COVID-19 infection clinically, although the pathophysiological distinction between the two remains to be fully elucidated. Lung function decline after COVID-19 tends to improve spontaneously [8]. In contrast, RAS progresses in a “stair-step” pattern characterized by multiple episodes of acute exacerbation [16]. In the present case, the patient had a fair prognosis for a long time after receiving LTx; however, within approximately four months after COVID-19 infection, lung volume loss and pulmonary respiratory failure advanced, and the patient died without the benefit of immunosuppressive therapy. Although COVID-19 pneumonia was well treated and ground-glass opacity and consolidation improved on CT with the negative conversion of SARS-CoV-2 PCR test, dyspnea became worse about eight weeks after COVID-19 infection. Radiologically, worsening of the transplant lung permeability and mediastinal deviation on chest x-ray, new ground-glass nodules, consolidation with traction bronchiectasis on CT, strong contractile changes, and lung volume loss appeared. We were unable to conduct a spirometry test due to the patient's compromised general health, resulting in the patient not meeting the definitive physiological criteria for CLAD, but the progressive “stair-step” decline in lung function accompanied by imaging abnormalities with typical imaging findings strongly suggested RAS. Hence, the conjecture regarding the pathophysiology of RAS following COVID-19 necessitates the accumulation of future cases, which may contribute to elucidating CLAD pathogenesis.

## Conclusions

Here, we presented a case of RAS following COVID-19 pneumonia. CLAD occurrence after COVID-19 disease has been suggested to be more likely to develop RAS than BOS. When evaluating chest images after pneumonia, clinicians should be alert for the appearance of image features suspicious of RAS and BOS. Knowledge of the imaging findings of CLAD (BOS and RAS) allows the detection of abnormalities at an early stage and increases the possibility of re-transplantation, which is the only effective treatment for CLAD.
